# Mechanism of anti-*Vibrio* activity of marine probiotic strain *Bacillus pumilus* H2, and characterization of the active substance

**DOI:** 10.1186/s13568-017-0323-3

**Published:** 2017-01-17

**Authors:** Xi-Yan Gao, Ying Liu, Li-Li Miao, Er-Wei Li, Ting-Ting Hou, Zhi-Pei Liu

**Affiliations:** 1 State Key Laboratory of Microbial Resources, Institute of Microbiology, Chinese Academy of Sciences, No. 1 West Beichen Road, Chaoyang District, Beijing, 100101 People’s Republic of China; 2 University of Chinese Academy of Sciences, Beijing, 100049 People’s Republic of China; 3 State Key Laboratory of Mycology, Institute of Microbiology, Chinese Academy of Sciences, Beijing, 100101 People’s Republic of China

**Keywords:** Anti-*Vibrio*, *Bacillus pumilus* H2, Mechanism, Amicoumacin A, Vibriosis control

## Abstract

Vibriosis is a major epizootic disease that impacts free-living and farmed fish species worldwide. Use of probiotics is a promising approach for prevention of *Vibrio* infections in aquaculture. A probiotic anti-*Vibrio* strain, *Bacillus pumilus* H2, was characterized, and the mechanism of its effect was investigated. All 29 *Vibrio* strains tested were growth-inhibited by H2. The anti-*Vibrio* substance present in cell-free supernatant of H2 was purified and characterized by reversed-phase HPLC. Minimum inhibitory concentrations of the purified substance, determined in liquid media for various *Vibrio* strains, ranged from 0.5 to 64 µg/ml. Addition of the purified substance to *Vibrio vulnificus* culture inhibited cell growth (estimated by OD_600_). Confocal microscopy and scanning electron microscopy analyses showed that surface structure of *V. vulnificus* cells was damaged by the purified substance, as reflected by presence of membrane holes, disappearance of cellular contents, and formation of cell cavities. The major mechanism of this anti-*Vibrio* activity appeared to involve disruption of cell membranes, and consequent cell lysis. The purified anti-*Vibrio* substance was shown to be structurally identical to amicoumacin A by MS and NMR analysis. Our findings indicate that *B. pumilus* H2 has strong potential for prevention or treatment of fish vibriosis in the aquaculture industry.

**Electronic supplementary material:**

The online version of this article (doi:10.1186/s13568-017-0323-3) contains supplementary material, which is available to authorized users.

## Introduction

During the course of aquaculture development, major production problems have been caused by a number of bacterial diseases (Paillard et al. 2004; Stentiford et al. 2012; Toranzo et al. 2005). These disease-related problems are the largest single cause of economic losses in aquaculture (Stentiford et al. 2012; Zhou et al.^2009^). A small number of opportunistic bacterial pathogens are responsible for the majority of such losses worldwide (Austin and Austin 2007). The Gram-negative genus *Vibrio* is one of the most important groups of bacterial pathogens, and a major source of mortality (Colwell and Griems 1984; Egidius 1987; Li and Woo 2003). *Vibrio* species are widespread and ubiquitous in aquatic environments worldwide, occupy a variety of habitats in marine, freshwater, and estuarine ecosystems, and are frequently found in aquaculture facilities (Heidelberg et al. 2002; Tall et al. 2013; Thompson et al. 2004).

Vibriosis, a collective *Vibrio* infection (Egidius 1987), is a widespread epizootic disease that affects most free-living and farmed fish species worldwide, and is currently the major limiting factor in development of intensive mariculture industry (Egidius 1987). In association with the rapid expansion of intensive mariculture and consequent deterioration of culture conditions, a steadily increasing number of *Vibrio* species are recognized as pathogens in vibriosis outbreaks (Austin and Zhang 2006; Cui et al. 2014; Hou et al. 2016). A limited number of antibiotics have been successfully applied, and resistance to these antibiotics may reduce the success of treatment programs (Al-Othrubi et al. 2014; Elmahdi et al. 2016).

The term “probiotic” was introduced by Parker in 1974, referring to “organisms and substances that have a beneficial effect on the host animal by contributing to its intestinal microbial balance” (Parker 1974). Many groups have investigated the benefits of using probiotic strains in aquaculture (Balcázar et al. 2006; Desriac et al. 2010; Moriarty 1998; Newaj-Fyzul et al. 2014; Verschuere et al. 2000). Species and strains of *Bacillus*, a genus of Gram-positive, rod-shaped bacteria, exert antagonistic or inhibitory activities against a variety of bacterial and fungal pathogens, and have been utilized frequently as probiotics for treatment and/or prevention of infectious processes in many plants and animals (Mongkolthanaruk 2012; Mondol et al. 2013; Patel et al. 2009; Wulff et al. 2002).

In previous study of our lab, probiotic effect of *Bacillus pumilus* H2 to juvenile shrimp was carried out in aquaculture tanks (Fu et al. 2009). Juvenile shrimp were exposed to *B. pumilus* H2 at 0 (as control), 10^3^ and 10^4^ CFU/ml for 14 days before a challenge with *Vibrio natriegens* at 10^4^ CFU/ml for 1 day infection. The final mortality of the shrimp group treated with 10^4^ CFU/ml *B. pumilus* H2 was only 12.5%, much lower than the group treated with 10^3^ CFU/ml *B. pumilus* H2 (28.3%) and the control group (30.8%, *P* < 0.05); and the average weight and length of the shrimp group treated with 10^4^ CFU/ml *B. pumilus* H2 were also higher than those of the control group (Fu et al. 2009). And results showed that H2 might have good application prospects and significance.

In the present study, we: (1) further screened *Bacillus* strains that displayed sufficient anti-*Vibrio* activity to be considered as biocontrol agents, (2) measured in vitro antagonistic activity of probiotic strain *B. pumilus* H2 against *Vibrio* species, and (3) extracted and purified antimicrobial compounds from H2, and made preliminary studies of their inhibitory mechanisms. The major of anti-*Vibrio* mechanism of H2 appeared to be disruption of the cell membrane, and the active anti-*Vibrio* compound was structurally identified as amicoumacin A. Our findings indicate that H2 has strong potential application in prevention or control of fish vibriosis.

## Materials and methods

### Bacterial strains and culture conditions

Bacterial strains used in this study included 29 *Vibrio* species, four *Bacillus* species, and two *Aeromonas* species (Table 1). All strains were confirmed by sequencing of their 16S rRNA gene. All *Vibrio* and *Aeromonas* species were used as target strains (indicator strains). Strains were recovered from a lyophilized ampoule or frozen stocks for 36 h aerobic incubation in liquid LB medium before use, and they were grown in LB medium or on LB plates at 30 °C under aerobic conditions.

**Table 1:** Bacterial strains used in this study

Genus	Species and strain	Source(s)	Date of collection
*Vibrio*	*V. vulnificus* CZ-A2, *V. diazotrophic* CZ-G1, *V. ponticus* CZ-L7, *V. neptunius* CZ-D1, *V. rotiferianus* CZ-F1, *V. sinaloensis* PE7, *V. communis* J7, *V. azureus* D3, *V. scophthalmi* E3, *V. chagasii* T3, *V. campbellii* AF5, *Vibrio ponticus* B8	Biofilters, fish ponds of marine aquaculture recirculating system	Collected by our lab in 2011
	*V. algoinfesta* QBST8, *V. alfacsensis* QBST3, *V. alginolyticus* LM3-1, *V. sinaloensis* QBSM3, *V. cyclitrophicus* DFWB3, *V. fortis* QBLM3, *V. owensii* QBST1, *V. ponticus* W6-3, *V. harveyi* LM2, *V. rotiferianus* W5-3	Skin, liver, and spleen of diseased marine aquaculture animals	Collected by our lab in 2013
	*V. alginolyticus* CGMCC 1.1607, *V. parahaemolyticus* CGMCC 1.2164, *V. fischeri* CGMCC 1.1613	China General Microbiological Culture Collection Center (CGMCC)	Bought from CGMCC in 2013
*Bacillus*	*B.* *pumilus* H2 (CGMCC No. 1004), *B.* *safensis* H2-2 (CGMCC No. 1006)	Marine sediment	Collected by our lab in 2005
	*B.* *velezensis* V4 (CGMCC No. 10149)	Marine aquaculture pond	Collected by our lab in 2011
	*B.* *methylotrophicus* L7	Stored in lab	Collected by our lab in 2011
*Aeromonas*	*A. hydrophila* CGMCC 1.0927	CGMCC	Bought from CGMCC in 2013
	*A. salmonicida* E11I4	Diseased marine fish	Collected by our lab in 2014

### Preparation of cell suspension of indicator strains

Indicator strains were inoculated in LB broth, incubated 24 h at 30 °C with shaking (150 rpm), and optical density at 600 nm (OD_600_) was determined. Cell suspensions of indicator strains were obtained by adjusting OD_600_ to 0.8 using sterile LB broth.

### Preparation of cell-free supernatant (CFS) of *Bacillus* strains

*Bacillus* strains were inoculated and incubated as above, and cells were removed by centrifugation (8000×*g*) for 10 min at 4 °C. Supernatants were passed through sterile syringe filters to obtain CFS.

### Screening and characterization of anti-*Vibrio* strains

Two approaches were used for screening of *Bacillus* strains having anti-*Vibrio* activity: (1) A given *Bacillus* strain was inoculated as a spot (diameter ~2–3 mm) on the surface of a LB agar plate spread with cell suspension of a given indicator strain. Cells were incubated 48 h at 30 °C, and antagonistic activity was evaluated based on the presence of a growth inhibition zone around the spot. (2) 10 µl CFS was dropped onto a 6-mm paper disk on an agar plate spread with cell suspension of a given indicator strain, and incubated 24 h at 30 °C. Anti-*Vibrio* activity was assessed as diameter (mm) of the inhibition zone between the disk and the bacterial lawn.

*Aeromonas salmonicida* E11I4 and *A. hydrophila* CGMCC 1.0927 were tested as reference strains

### Extraction and purification of antimicrobial compounds

Strain H2 was inoculated on three 200 ml LB broth for 24 h using shaking flasks (150 r/min) at 30 °C. 600 ml CFS in total was lyophilized (Heto PowerDry PL6000, Thermo Scientific, USA), lyophilized material was extracted with methanol, and dried methanol extract was dissolved in 20 mM Tris–HCl (pH 7.0) and applied to a solid-phase extraction (SPE) column (Bond Elut C18, Varian, USA) to remove impurities. Fractions (each 10 ml) were eluted from the SPE column by acetonitrile concentration gradient (0, 10, 20, 30, 40% acetonitrile in H_2_O), and anti-*Vibrio* activity of each fraction was tested using *V. vulnificus* as indicator. Active fractions were pooled, lyophilized, and further purified by reversed-phase high performance liquid chromatography (RP-HPLC). A C18 semi-preparative column (Zorbax SB-C18, 5 µm, 9.4 × 150 mm, Agilent) was developed with gradient 20% acetonitrile/0.1% trifluoroacetic acid (TFA) in H_2_O to 40% acetonitrile/0.1% TFA in H_2_O, from 5 to 42 min, at flow rate 2 ml/min. Anti-*Vibrio* activity of each collected peak was assessed using *V. vulnificus* as indicator. The active peak was identified at 16 min, which corresponds to acetonitrile concentration 34%. Purified anti-*Vibrio* substance was obtained by lyophilization of this fraction.

### Determination of minimum inhibitory concentration (MIC)

MICs of purified antimicrobial substances from various *Vibrio* strains were determined by broth microdilution assays in 96-well microwell plates. 100 µl of twofold serial dilutions of purified anti-*Vibrio* substance was mixed with an equal volume of 1:100-diluted overnight *Vibrio* cultures in sterile LB. Negative control wells (without purified anti-*Vibrio* substance) and wells containing only LB were included in the assay. Plates were incubated 24 h at 30 °C. Concentration of colony-forming units (CFUs) in the bacterial inoculum was ~10^5^ CFU/ml. MIC was defined as the lowest concentration of antimicrobial substance that completely inhibited bacterial growth.

### Confocal microscopy

*Vibrio vulnificus* cells grown for 24 h were incubated 24 h with anti-*Vibrio* substance (final concentration 0.5, 5, or 10 µg/ml), or with PBS alone. SYTOX green (SG) (Molecular Probes, Invitrogen, USA) was then added (final concentration 0.8 µM), and samples were incubated 15 min in the dark. Cells were washed, resuspended in phosphate buffer saline (PBS), prepared as confocal slides, and visualized by confocal microscopy. Fluorescence was photographed with a fluorescence microscope (model CTR 5000, Leica, Germany), with filters set at excitation wavelength 488 nm/emission wavelength 538 nm, for SG detection.

### Scanning electron microscopy (SEM)

*Vibrio vulnificus* cells grown for two days in LB were incubated 24 h with anti-*Vibrio* substance (0.5 µg/ml), with sterile PBS (pH 7.2) as control. Cells were resuspended in 2.5% (v:v) glutaraldehyde solution in 0.1 M PBS, and fixed for 24 h. The glutaraldehyde was removed, and 1% osmium tetroxide solution (pH 7.2) was added. After 1.5 h, cells were washed three times with PBS. Cells were then (1) dehydrated by cold ethanol concentration gradient (10, 30, 50, 70, 90%; 10 min each), and (2) dehydrated twice in 100% ethanol at 10 min intervals. For SEM assay, cells were washed with 50, 70, 90, and 100% isoamyl acetate (each 3 min), critical point dried, coated with gold/palladium, and observed and photographed with a scanning electron microscope (model S-3400N, Hitachi Instruments, Japan).

### Structure determination of antimicrobial compound

For mass spectrometer (MS) analysis, purified anti-*Vibrio* substance was dissolved in 30% acetonitrile in H_2_O and injected into an Orbitrap Fusion mass spectrometer (Thermo-Fisher, USA). For nuclear magnetic resonance (NMR) analysis, purified anti-*Vibrio* substance (5 mg) was dissolved in 200 μl dimethyl sulfoxide (DMSO), and samples were pipetted into a DMSO-matched NMR tube (Shigemi Co., Japan) for NMR analysis (model Avance III, 500 MHz, Bruker, USA).

## Results

### Screening of probiotic *Bacillus* strains

Four *Bacillus* strains (*B. velezensis* V4, *B. methylotrophicus* L7, *B. pumilus* H2, *B. safensis* H2-2) were screened for anti-*Vibrio* activity. Each of the four strains had growth-inhibiting effects on various *Vibrio* strains. *B. pumilus* H2 had the broadest anti-*Vibrio* activity spectrum; it inhibited all 29 *Vibrio* strains tested to varying degrees (Additional file 1: Table S1). When CFS of H2 was tested, the diameter of its inhibition zone for the *Vibrio* strains ranged from 7 to 18 mm. When supernatant was concentrated, the inhibition zone diameters became significantly larger (17 to 25 mm). We therefore selected H2 as the probiotic strain used in further studies of anti-*Vibrio* activity.

### Growth curve and anti-*Vibrio* activity of *B. pumilus* H2

**Fig. 1:**
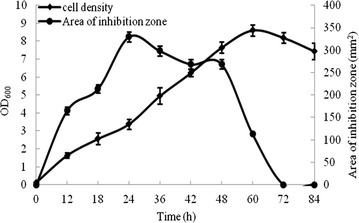
Growth curve and anti-*Vibrio* activity of *B. pumilus* strain H2

H2 accumulated anti-*Vibrio* substance in culture broth before 24 h. As incubation continued, anti-*Vibrio* activity declined, and was essentially gone after 3–4 days (Fig. 1).

### Effects of enzymes, heat, pH, and chemicals on anti-*Vibrio* activity

We performed a series of stability assays to gather information on chemical structure of the anti-*Vibrio* substance, and reference data for its practical application as a probiotic.

The results (Additional file 1: Table S2) indicated that the anti-*Vibrio* substance presented in the CFS was very thermal stable, there was 69.73% activity remained after being treated at 121 °C for 15 min, in comparison with the control (−20 °C, 60 min). The anti- *Vibrio* substance also performed quite well in resistance to enzyme digestion, since none of the enzymes tested (proteinase K, trypsin, chymotrypsin, lysozyme) caused complete disappearance of activity. The results also showed that organic solvents only slightly affected the anti-*Vibrio* substance, most of the activity (80–90% relative activity) was remained after being treated with addition of equal volume of organic solvents at 37 °C for 1 h. Activity was maintained over a wide range of pH values, from 2 to 10. UV irradiation had little effect on activity, there was only a 12% reduction even after exposure to UV at a distance of 25 cm for 5 h (Additional file 1: Table S2).

### Extraction and purification of anti-*Vibrio* substance

**Fig. 2:**
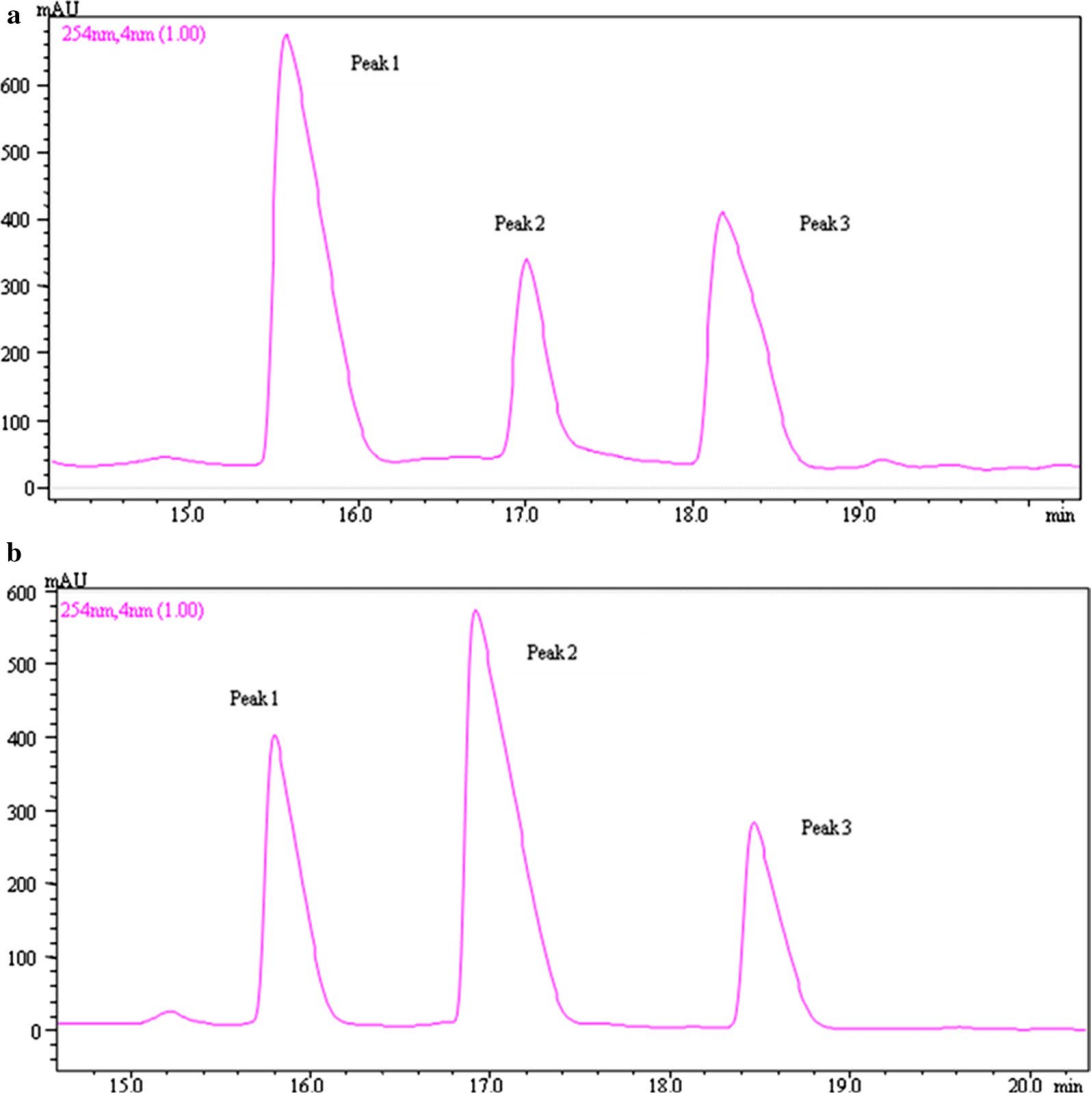
Spectra of crude extract after purification by SPE and RP-HPLC, from 24 h-CFS (**a**) and 36 h-CFS (**b**)

The anti-*Vibrio* substance present in *B. pumilus* H2 CFS was purified by SPE and RP-HPLC. The HPLC spectrum showed three peaks (Fig. 2). In anti-*Vibrio* activity assays using *V*. *vulnificus* CZ-A2 as target organism, activity was strong for peak 1 and very weak for peak 2, suggesting that the peak 1 substance was the major anti-*Vibrio* compound. In CFS cultured for 24 h, peak 1 was dominant, whereas in CFS cultured for 36 h peak 2 was dominant one and peak 1 declined greatly. This observation is consistent with the activity curve associated with H2 growth (Fig. 1).

A total of 20 mg purified anti-*Vibrio* substance was obtained by semi-preparative RP-HPLC and used for subsequent experiments.

### MICs of purified anti-*Vibrio* substance for various *Vibrio* strains

MICs of purified anti-*Vibrio* substance for various *Vibrio* strains, determined in liquid media, ranged from 0.25 to 64 μg/ml (Table 2). The purified substance showed high inhibitory activity against *V. natriegens* FS-1, *V. vulnificus* CZ-A2, *V. harveyi* PH4, *V*. *sinaloensis* PE7, and *V*. *ponticus* B8, but less activity against *V*. *diazotrophicus* CZ-G1, *V*. *alginolyticus* CGMCC 1.1607, and *V*. *parahaemolyticus* CGMCC 1.2164.

**Table 2:** MICs of purified anti-*Vibrio* substance for various *Vibrio* strains

MIC (µg/ml)	Strain
0.25	*Vibrio natriegens* FS-1
0.5	*V. vulnificus* CZ-A2, *V. harveyi* PH4, *V*. *sinaloensis* PE7, *V*. *ponticus* B8
2	*V*. *alfacsensis* QBST3, *V*. *communis* J7
4	*V*. *azureus* D3, *V*. *algoinfesta* QBST8, *V*. *fortis* QBLM4
8	*V*. *alginolyticus* LM3-1, *V*. *cyclitrophicus* DFWB3, *V*. *scophthalmi* E3, *V*. *fortis* QBLM3, *V*. *owensii* QBST1, *V*. *ponticus* W6-3, *V*. *rotiferianus* CZ-F1, *V*. *rotiferianus* W5-3
16	*V*. *neptunius* CZ-D1, *V*. *sinaloensis* QBSM3, *V*. *campbellii* AF5, *V*. *harveyi* LM2, *V*. *fischeri* CGMCC 1.1613
32	*V*. *ponticus* CZ-L7, *V*. *chagasii* T3, *V*. *anguillarum* XP
64	*V*. *diazotrophic* CZ-G1, *V*. *alginolyticus* CGMCC 1.1607, *V*. *parahaemolyticus* CGMCC 1.2164

### Effect of purified anti-*Vibrio* substance on growth of *V. vulnificus*

**Fig. 3:**
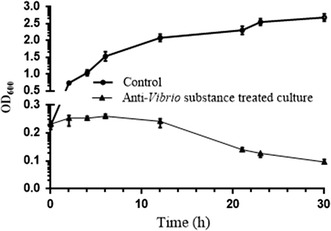
Effect of purified anti-*Vibrio* substance on growth of *V. vulnificus*

*Vibrio vulnificus* was selected as a model target strain for experiments on the mode of interaction of purified anti-*Vibrio* substance with *Vibrio* strains. *V. vulnificus* was inoculated into LB broth (100 ml) to OD_600_ ~0.25, and purified anti-*Vibrio* substance was added (control = sterile distilled water). Growth was monitored by measuring OD_600_ of the culture at predetermined intervals. Growth of the target strain was clearly inhibited by addition of anti-*Vibrio* substance; i.e., OD_600_ did not increase as it did in control culture (Fig. 3). OD_600_ declined continuously after 8 h incubation, suggesting that cell lysis was occurring. In the control group, the target strain showed exponential growth immediately after inoculation (Fig. 3).

### Effect of purified anti-*Vibrio* substance on cell surface structure of *V. vulnificus*

Membrane integrity of *V. vulnificus* (target strain) following treatment with purified anti-*Vibrio* substance was evaluated by confocal microscopy and an assay based on uptake of the fluorescent dye SG. SG, a high-affinity nucleic acid stain, is often used to assess integrity of plasma membranes, because it easily penetrates cells with compromised membranes, but does not pass through membranes of non-compromised cells.

**Fig. 4:**
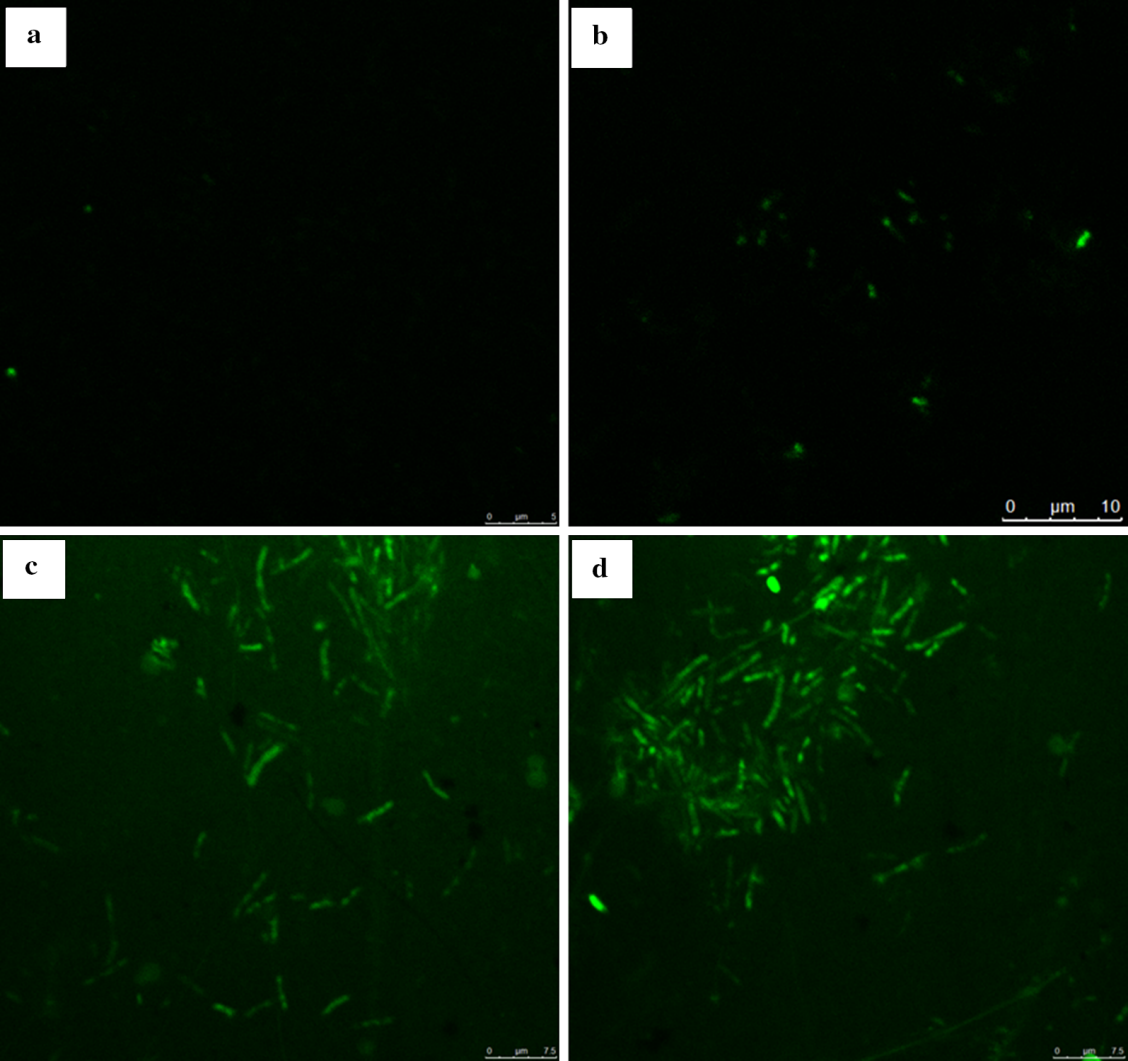
Confocal microscopic images of *V. vulnificus* cells treated with purified anti-*Vibrio* substance at 0 μg/ml (**a**), 0.5 μg/ml (**b**), 5 μg/ml (**c**), and 10 μg/ml (**d**)

Nontreated *V. vulnificus* cells (control) showed no appreciable fluorescent signal (Fig. 4). Treatment with 0.5 μg/ml purified substance resulted in detection of only a very weak fluorescent signal. Intensity of the fluorescent signal increased steadily as substance concentration increased. At substance concentration 10 µg/ml, the fluorescent signal was distinct, clear, and strong, indicating that the cell membrane was completely permeabilized.

**Fig. 5:**
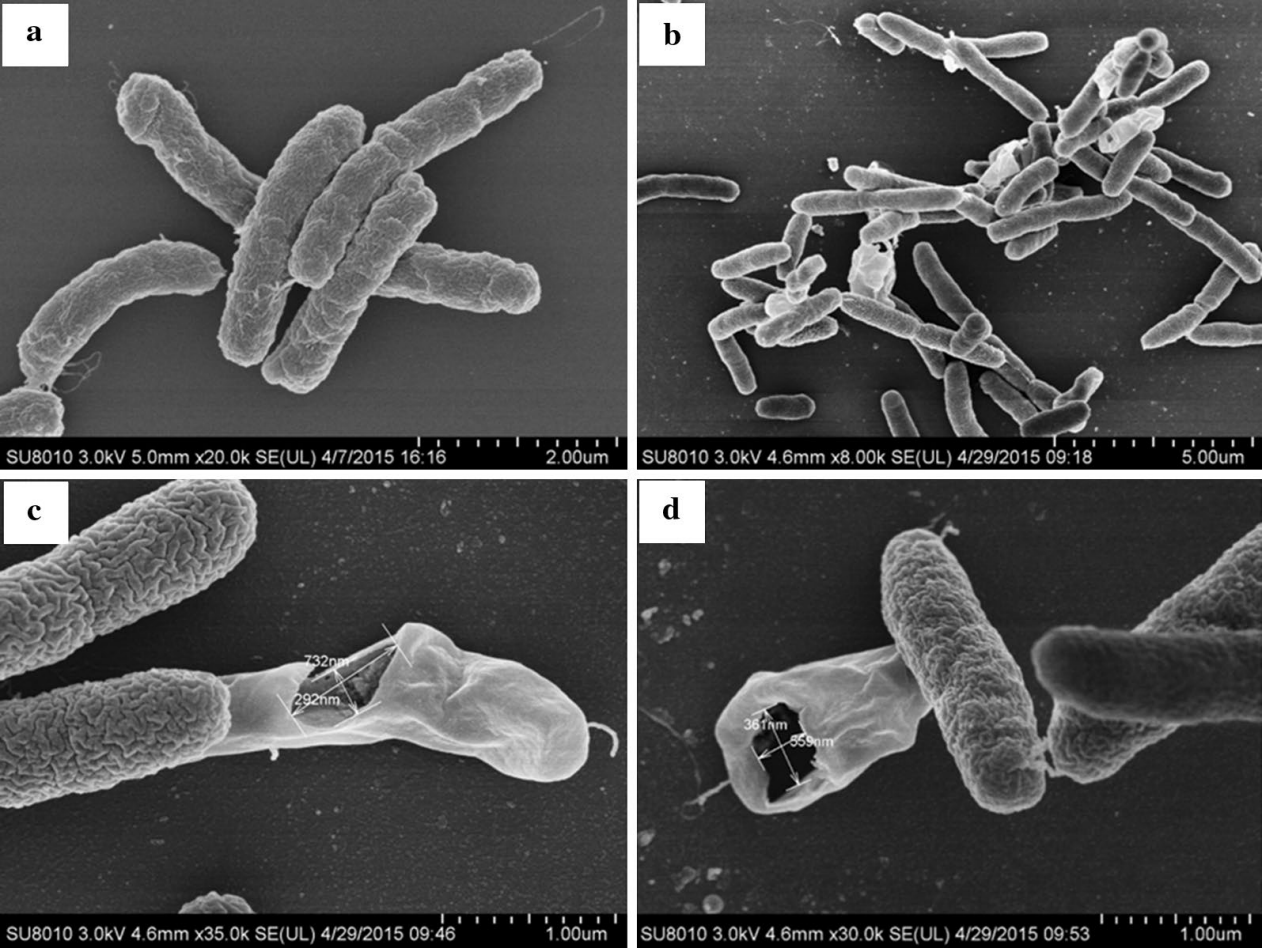
SEM images of *V. vulnificus* CZ-A2 cells treated with purified anti-*Vibrio* substance at 0 μg/ml (**a**, 20,000×; control) and 0.5 μg/ml (**b**, 8000×;** c**, 35000×;** d**, 30,000×), showing formation of membrane holes

Changes in surface structure of *V. vulnificus* cells resulting from treatment with purified anti-*Vibrio* substance were analyzed by SEM. Nontreated cells were intact, smooth, and displayed fine structure (Fig. 5a). In contrast, cells treated with the substance showed clear surface structure damage, including appearance of membrane holes, disappearance of cellular contents, and formation of cell cavities (Fig. 5b–d). The size of these cavities (292 × 732 nm in Fig. 5c; 361 × 559 nm in Fig. 5d) indicated that cell lysis had occurred.

### Structural characterization of purified anti-*Vibrio* substance

**Fig. 6:**
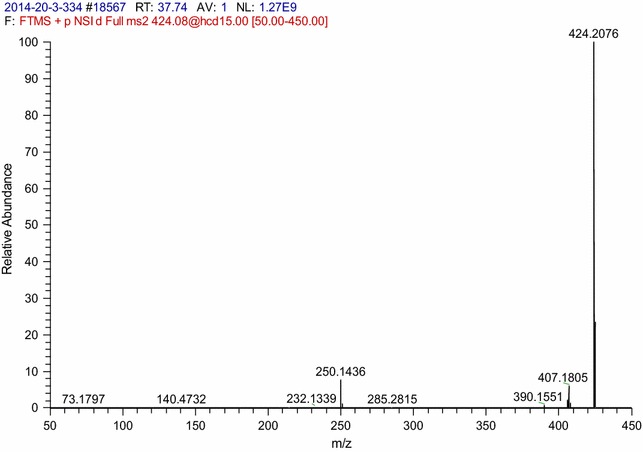
MS analysis of purified anti-*Vibrio* substance from HPLC peak 1 in Fig. 2

The molecular mass of the purified anti-*Vibrio* substance (peak 1 in Fig. 2) was 423.2076 Da, as determined by Orbitrap Fusion MS. The MS analysis also revealed a plausible chemical formula (C_20_H_30_N_3_O_7_) for the substance (Fig. 6). Chemical shifts and coupling constants were assigned to protons in the molecule through NMR analysis. The ^13^C-NMR spectra of the purified anti-*Vibrio* substance (peak 1 in Fig. 2) showed twenty signals at 21.7 (q), 23.6 (q), 25.2 (d), 30.0 (t), 32.3 (t), 39.1 (t), 50.2 (d), 51.2 (d), 71.2 (d), 73.2 (d), 82.1 (d), 108.6 (s), 170.5 (s), 173.8 (s) and 175.1 (s) ppm, respectively. Comparisons of molecular mass, chemical formula, and NMR data with those of compounds previously described in the literature indicated that the purified anti-*Vibrio* substance is identical to amicoumacin A (Itoh et al. 1981). Two structurally related substances (peaks 2 and 3 in Fig. 2) were also characterized. Based on MS analysis, peak 2 ((M + H)^+^ ion at m/z 425 Da) was identified as amicoumacin B (C_20_H_28_N_2_O_8_), and peak 3 ((M + H)^+^ ion at m/z 407 Da) was identified as amicoumacin C (C_20_H_26_N_2_O_7_) (data not shown).

## Discussion

As the aquaculture industry expands worldwide, and the variety of fish species involved increases, many unknown fish-pathogenic *Vibrio* species are reported (Li and Woo 2003; Thompson et al. 2004; Austin and Zhang 2006; Cui et al. 2014). In the past, vibriosis was controlled (prevented or treated) almost exclusively through application of antibiotics or chemotherapeutic agents, either as feed additives or in immersion baths. However, extensive use of this approach over time has resulted in increased resistance of pathogenic *Vibrio* strains to the commonly used antibiotics: ampicillin, amikacin, kanamycin, penicillin G, streptomycin, and tetracycline (Austin and Austin 2007; Li et al. 1999; Elmahdi et al. 2016).

In present study, we screened *Bacillus* strains for anti-*Vibrio* activity. *Bacillus* strains are good candidates as biological control agents for prevention or treatment of plant and animal infections for several reasons (Wulff et al. 2002; Mongkolthanaruk 2012; Mondol et al. 2013). (1) They produce antibiotics having well-documented antagonistic activity against a variety of fungal and bacterial pathogens. (2) They form spores that can be easily formulated, and have high viability in comparison with vegetative cells. (3) The robustness of the spores enables them to cross the gastric barrier. A certain proportion of spores is thus able to germinate in and colonize (albeit briefly) the intestinal tract (Mazza 1994). (4) *Bacillus* species are abundant in a wide variety of environments and habitats.

*Bacillus pumilus* strain H2 has notable anti-*Vibrio* effects. It inhibited 29 *Vibrio* strains to varying degrees (Table 2). No anti-*Vibrio* probiotic has been previously reported to inhibit such a large number of *Vibrio* strains. *V. vulnificus* CZ-A2, *V. natriegens* FS-1, *V. harveyi* PH4, *V. sinaloensis* PE7, *V. ponticus* B8, *V. alfacsensis* QBST3, and *V. communis* J7 were highly sensitive to purified anti-*Vibrio* substance. *V. anguillarum* is the most well-studied and widespread fish-pathogenic *Vibrio* species, and is responsible for the majority of fish loss in aquaculture worldwide (Austin and Austin 2007). We measured MIC of purified anti-*Vibrio* substance from H2 against *V. anguillarum* XP as 32 µg/ml, indicating its potential application for control of this major pathogen.

We selected *V. vulnificus* CZ-A2 as the target strain for screening of *Bacillus* strains having anti-*Vibrio* activity, and for follow-up studies of the mechanism of H2 activity, because CZ-A2 was highly sensitive to the substance present in H2 CFS. *V. vulnificus* is a widespread marine bacterium categorized into three biotypes. Strains of *V. vulnificus* include one of the most widely occurring fish pathogens, and another strain that can cause wound infections in humans, resulting in high mortality among susceptible individuals (Efimov et al. 2013; Ziolo et al. 2014).

The anti-*Vibrio* substance produced by *B. pumilus* H2 was found to be structurally identical to amicoumacin A, which was described in 1981 (Itoh et al. 1981). Amicoumacin A and related compounds display inhibitory activity against numerous pathogenic bacteria, including *Helicobacter pylori* and methicillin-resistant *Staphylococcus aureus* (MRSA) (Pinchuk et al. 2001; Lama et al. 2012). Anti-inflammatory and antitumor effects of amicoumacin A have also been reported (Itoh et al. 1981), but no study to date has addressed its effects against pathogenic *Vibrio* that cause economic losses in aquaculture. Amicoumacin B was isolated from *B. pumilus* and reported to display gastroprotective activity, but weak antibacterial and weak antiulcer activity (Shimojima et al. 1984; Han et al. 2013), consistent with the weak anti-*Vibrio* activity that we observed. Activity of amicoumacin C has not been studied. Although application of H2 preparation was used and showed no toxic to juvenile shrimp, further tests on toxic of purified Amicoumacin A to one or more farmed species need to be conducted in the future.

The mode of action of amicoumacin A remains unclear. Lama et al. reported amicoumacin A-induced alteration of transcription of genes that regulate various cellular processes, including cell envelope turnover, cross-membrane transport, virulence, metabolism, and general stress. The gene most highly induced by amicoumacin A was *lrgA*, which encodes an antiholin-like product (LrgA) that appears in cells undergoing collapse of Δψ, and modulates murein hydrolase activity (Lama et al. 2012). Taken together, the findings of Lama et al. suggest that amicoumacin A provokes perturbation of the cell membrane and consequent energy dissipation (Lama et al. 2012). Polikanov et al. proposed that amicoumacin A is a potent inhibitor of protein synthesis, but without direct experimental evidence (Polikanov et al. 2014). Our observations of reduced cell density (Fig. 3), formation of membrane holes, disappearance of cellular contents, and formation of cell cavities (Figs. 4, 5) indicates that the major mechanism of amicoumacin A activity against pathogens involves disruption of cell membranes, and consequent cell lysis.

*Bacillus pumilus* H2 was isolated from marine sediment, and therefore has priority and inherent advantages for use in marine aquaculture as a biocontrol agent or probiotic. Under the generally accepted definition of the term “probiotic”, we can consider two application approaches. In the first approach, H2 fermentation broth would be added, in proportion, directly to the aquaculture pond. Live H2 cells would then produce amicoumacin A continuously. In simulated gastroenteric environments designed to test spore robustness, H2 spores were able to cross the gastric barrier (data not shown), and may therefore have the ability to germinate and colonize in the intestinal tract. Probiotic effects in aquaculture are not limited to the intestinal tract, but may also improve the health of the host by inhibiting pathogens and improving water quality through modification of microbial community composition in the water and sediment (Perez-Sanchez et al. 2013).

In the second approach, extracted amicoumacin A would be added to aquaculture feeds. Amicoumacin A is potentially suitable for this purpose because it is heat stable, pH stable, UV stable, and not sensitive to various enzymes and organic solvents. On the other hand, a major disadvantage of the second approach is that amicoumacin A is difficult to extract and purify. The first approach appears more feasible.

In conclusion, probiotic *B. pumilus* strain H2 demonstrated notable antagonistic activity against 29 *Vibrio* strains tested. This activity was attributable to production of amicoumacin A, which has been reported previously to inhibit methicillin-resistant *Staphylococcus aureus* and *Helicobacter pylori*, and to display anti-inflammatory and antitumor effects. The major mechanism of amicoumacin A activity against pathogens involves disruption of cell membranes, and consequent cell lysis.

## Additional file

**Additional file 1.** Additional tables.

## Abbreviations

CFS: cell-free supernatant
SPE: solid-phase extraction
RP-HPLC: reversed-phase high performance liquid chromatography
MIC: minimum inhibitory concentration
CFUs: colony-forming units
SG: SYTOX green
PBS: phosphate buffer saline
DMSO: dimethyl sulfoxide
MS: mass spectrometer
NMR: nuclear magnetic resonance
SEM: scanning electron microscope
MRSA: methicillin-Resistant *Staphylococcus aureus*
