# Supplementary material for: Mechanism of anti-Vibrio activity of marine probiotic strain Bacillus pumilus H2, and characterization of the active substance
Source: AMB Express. 2017 Jan 17;7:23. doi: 10.1186/s13568-017-0323-3 (PMC5241254; doi:10.1186/s13568-017-0323-3)
Supplement: Supplementary file 1 — Additional file 1. Additional tables. [file 13568_2017_323_MOESM1_ESM.doc]

AMB Express

Supplementary materials for

**Mechanism of anti-*Vibrio* activity of marine probiotic strain *Bacillus pumilus* H2, and characterization of the active substance**

Xi-Yan Gao^1, 2^ (gaoxiyan88@126.com), Ying Liu^1^ (liuying@im.ac.cn), Li-Li Miao^1^ (miaoll@im.ac.cn), Er-Wei Li^3^ (liew@im.ac.cn), Ting-Ting Hou^1^ (zhuyeqingting@yeah.net), Zhi-Pei Liu^1*^ (liuzhp@im.ac.cn)

^1^ State Key Laboratory of Microbial Resources, Institute of Microbiology, Chinese Academy of Sciences, Beijing 100101, China.

^2^ University of Chinese Academy of Sciences, Beijing 100049, China.

^3^ State Key Laboratory of Mycology, Institute of Microbiology, Chinese Academy of Sciences, Beijing 100101, China.

* Correspondence: Institute of Microbiology, Chinese Academy of Sciences, No. 1 West Beichen Road, Chaoyang District, Beijing 100101, P. R. China, Tel: +86-10-64806081

**Supplementary Tables**

**Table S1.** Inhibitory spectrum of *Bacillus* species.

| **Antagonistic strain** | **Inhibitory spectrum** |
| --- | --- |
| *Bacillus* *pumilus* H2 | *V. vulnificus* CZ-A2, *V*.*ponticus* CZ-L7, *V*.*natriegens* FS-1, *V*. *neptunius* CZ-D1, *V*. *sinaloensis* QBSM3, *V*. *fortis* QBLM3,*V*. *harveyi* PH4,*V*. *alginolyticus* LM3-1, *V*. *ponticus* B8,*V*. *alginolyticus* 1.1607, *V*. *rotiferianus* CZ-F1,*V*. *ponticus* W6-3, *V*. *rotiferianus* W5-3 , *V*. *fortis* QBLM4, *V*.*alfacsensis* QBST3,*V*.*owensii* QBST1, *V*.*cyclitrophicus* DFWB3, *V*. *harveyi* LM2, *V*. *anguillarum* XP, *V*. *sinaloensis* PE7, *V*. *communis* J7, *V*. *algoinfesta* QBST8, *V*. *fischeri* 1.1613, *V*. *parahaemolyticus* 1.2164, *V*. *diazotrophic* CZ-G1, *V*. *campbellii* AF5, *V*. *chagasii* T3, *V*. *azureus* D3, *V*. *scophthalmi* E3, *Aeromonas salmonicida* E11I4 |
| *B. safensis* H2-2 | *V*. *vulnificus* CZ-A2, *V*.*ponticus* B8,  *V*.*natriegens* FS-1, *V*. *neptunius* CZ-D1 |
| *B. velezensis* V4 | *V*.*natriegens* FS-1, *V*.*vulnificus* CZ-A2,  *V*.*harveyi* PH4,*V*. *ponticus* B8, *Aeromonas hydrophila* 1.0927,*A. salmonicida* E11I4 |
| *B. methylotrophicus* L7 | *V*. *natriegens* FS-1,*V*.*harveyi* PH4 |

**Table S2**. Effects of heat, enzymes, solvents, pH, and UV irradiation on anti-*Vibrio* activity of CFS.

|  | Treatment | Relative activity* |
| --- | --- | --- |
| Thermal stability | -20 °C, 60 min (control) | 100% |
|  | 15 °C, 60 min | 97.94% |
|  | 30 °C, 60 min | 97.94% |
|  | 60 °C, 60 min | 64.70% |
|  | 80 °C, 60 min | 47.72% |
|  | 100 °C, 30 min | 69.40% |
|  | 100 °C, 60 min | 25.53% |
|  | 121 °C, 15 min | 69.73% |
| Sensitivity to enzymes | Control | 100% |
|  | Proteinase K | 65.8% |
|  | Trypsin | 84.76% |
|  | Chymotrypsin | 80.88% |
|  | Lysozyme | 84.76% |
| Sensitivity to organic solvents | Control | 100% |
|  | Ethyl ether | 91.67% |
|  | Acetone | 87.78% |
|  | Acetic ether | 76.47% |
|  | Methanol | 97.85% |
|  | Ethanol | 95.75% |
|  | Acetonitrile | 100% |
| pH stability | pH 2 | 65.34% |
|  | pH 3 | 65.34% |
|  | pH 4 | 91.67% |
|  | pH 5 | 95.75% |
|  | pH 6 | 95.74% |
|  | pH 7 | 100% |
|  | pH 8 | 65.34% |
|  | pH 9 | 55.48% |
|  | pH 10 | 43.53% |
|  | pH 11 | 21.90% |
|  | pH 12 | 0% |
| UV stability | control | 100% |
|  | UV - 1 h | 100% |
|  | UV - 3 h | 87.60% |
|  | UV - 5 h | 87.78% |

* Activity relative to control.
